# Hyperglycaemia in pregnancy: Outcomes and diagnostic accuracy of combined modalities

**DOI:** 10.1016/j.clinme.2025.100495

**Published:** 2025-08-06

**Authors:** Jaishil Manga, Natalie Odell, Lungile Khambule, Sayuri Harishun, Farzahna Mohamed

**Affiliations:** a Charlotte Maxeke Johannesburg Academic Hospital, Department of Internal Medicine, Faculty of Health Sciences, University of the Witwatersrand, Johannesburg, South Africa; b Charlotte Maxeke Johannesburg Academic Hospital, Department of Obstetrics & Gynaecology, Faculty of Health Sciences, University of the Witwatersrand, Johannesburg, South Africa; c Charlotte Maxeke Johannesburg Academic Hospital, Department of Chemical Pathology, Faculty of Health Sciences, University of the Witwatersrand, Johannesburg, South Africa; d Charlotte Maxeke Johannesburg Academic Hospital, Department of Internal Medicine, Division of Endocrinology and Metabolism, Faculty of Health Sciences, University of the Witwatersrand, Johannesburg, South Africa

**Keywords:** Gestational diabetes mellitus, Maternal medicine, Fasting plasma glucose, Oral glucose tolerance test, OGTT, Glycated haemoglobin, HbA1c

## Abstract

The prevalence of diabetes mellitus (DM) during pregnancy is rising globally, yet data on gestational diabetes mellitus (GDM) in South Africa remain limited. This study retrospectively analysed glycaemic characteristics and outcomes in 298 women with pre-gestational DM and hyperglycaemia first detected in pregnancy (HFDP) between August 2019 and January 2021. Hyperglycaemic disorders were attributed to GDM (39.6%, n = 118), followed by type 2 diabetes (T2DM) (29.2%, n = 87), overt DM (22.1%, n = 66) and type 1 diabetes (T1DM) (9.1%, n=27). Significant risk factors for GDM included family history, obesity, poor obstetric history, and a previous history of macrosomia in pregnancy. Glycated haemoglobin (HbA1c) levels at booking were highest in women with pre-gestational DM (8.8% in T1DM and 7.8% in T2DM). A combination of HbA1c ≥ 5.75% and fasting plasma glucose (FPG) of 5.1–6.9 mmol/L was the most accurate method for diagnosing GDM, with an area under the curve (AUC) of 0.93. Postpartum follow-up using an oral glucose tolerance test (OGTT) revealed that 21% of women with GDM developed DM, while 53% had an impaired glucose tolerance (IGT). However, only 48% of those with GDM were followed up postpartum, highlighting the significant challenge of loss to follow-up. These findings emphasise the growing prevalence of HFDP and the elevated risk of postpartum DM, highlighting the need for improved follow-up care to mitigate long-term complications. Additionally, the combination of FPG and HbA1c ≥ 5.75% shows promise for enhancing GDM diagnosis and screening protocols, particularly in developing countries. Further research is needed to validate these findings and assess their broader applicability.

## Highlights

• The global prevalence of diabetes mellitus in pregnancy is increasing, primarily due to the obesity epidemic, raising significant public health concerns in low- and middle-income countries.
• Maternal hyperglycemia poses short-term risks like pre-eclampsia and neonatal complications, and long-term risks including obesity and diabetes in mother and child, emphasising the need for early detection and optimal care.
• The combination of fasting plasma glucose and glycated haemoglobin should be further explored for diagnosing gestational diabetes mellitus (GDM), as dual testing could improve screening in developing countries.
• Clinical practice and policy implications include revising GDM screening and follow-up protocols in developing regions to ensure timely interventions, enhancing detection and management, and reducing complications for mothers and infants.

## Introduction

The global rise in diabetes mellitus (DM), projected to affect 629 million people by 2045, has contributed to increasing rates of gestational diabetes (GDM), affecting one in seven births globally and over 15% of pregnancies in South Africa, particularly in urban areas with high diabetes burdens.^1^^,^^2^ However, over the past four decades, only seven prevalence studies of hyperglycaemia first detected in pregnancy (HFDP) have been conducted, revealing a wide range from 1.8% to 25.8%.^3^ These discrepancies likely arise from varying screening strategies and diagnostic criteria, laboratory inaccuracies and biases in glucose measurements.^3^ Additionally, the true burden of HFDP is likely underestimated due to risk-based screening approaches, limited awareness among healthcare providers and the public, and a healthcare system that prioritises selective disease management over comprehensive care that includes prevention.^3^^,^^4^ These gaps highlight the need for improved strategies to identify and manage GDM in South Africa.

The World Health Organization (WHO) and the Society for Endocrinology, Metabolism and Diabetes of South Africa (SEMDSA) classify hyperglycaemia in pregnancy as either pre-gestational DM including type 1 (T1DM), type 2 (T2DM) and HFDP, which includes GDM or overt DM.^5^^,^^6^ GDM refers to glucose intolerance first recognised during pregnancy, which is not overt DM with resolution post-delivery.^5^^,^^6^ Although the WHO endorses universal screening for GDM, this remains controversial, with no consensus on the optimal gestational timing and modality. Low- and middle-income countries (LMICs) face challenges in implementing universal screening due to cost and accessibility.^7^ A single testing modality is desirable for diagnosis; however, OGTT has limitations, including poor reproducibility and complicated protocols. Fasting plasma glucose (FPG) is simple and reproducible, but excluding the 1- and 2-hour plasma glucose can overlook patients with normal initial FPG but abnormal post-glucose levels.^7^^,^^8^ HbA1c is the standard for assessing chronic glycaemic control, being rapid, cost-effective and convenient. However, its use as a single diagnostic modality remains controversial due to significant ethnic and physiological variations during pregnancy.^9^ Thus, there is growing interest in alternative and combined diagnostic modalities.

T2DM is often undetected until pregnancy, where it is mistakenly classified as GDM. Many women with GDM face an increased risk of developing T2DM, particularly as the obesity and ageing rates rise. South African women of reproductive age exhibit the highest obesity prevalence in sub-Saharan Africa, ranging from 15.9% to 67.8%.^10^ Emerging evidence indicates that hyperglycaemia during pregnancy can lead to significant maternal, fetal and neonatal complications, perpetuating metabolic issues for future generations.^10^

This study aims to evaluate the diagnostic utility of combining FPG and HbA1c in the detection of GDM, in comparison to the standard OGTT. Additionally, it seeks to characterise the glycaemic profiles and assess both maternal and neonatal outcomes among obstetric patients with pre-gestational DM and GDM. The study also investigates the incidence of postpartum dysglycaemia in this population. Addressing these critical gaps in antenatal screening and follow-up is essential to facilitate early diagnosis and intervention, ultimately promoting long-term metabolic health for both mothers and their offspring.

## Materials and methods

This retrospective single-centre study included antenatal patients with pre-gestational DM (T1DM or T2DM) and HFDP (overt DM and GDM) managed at the Combined Obstetric-Endocrine Diabetic Clinic at Charlotte Maxeke Johannesburg Academic Hospital (CMJAH) between August 2019 and January 2021.

Primary outcomes included the number of patients presenting with pre-gestational DM and HFDP and associated maternal and fetal outcomes. The secondary outcome assessed the diagnostic performance of combined FPG and HbA1c for GDM.

Patients with one or more GDM risk factors (Supplementary Table 1) underwent a 75 g OGTT per IADPSG guidelines.^11^ The OGTT involved sampling at fasting, 1 h and 2 h post-glucose ingestion. Glycaemic status was classified using the 2017 SEMDSA criteria (Supplementary Table 2).^5^^,^^6^ Overt DM was defined as HbA1c ≥ 6.5% or random glucose ≥11.1 mmol/L confirmed by repeat testing.^5^^,^^6^ SEMDSA recommends risk-based OGTT at booking, with repeat testing at 24–28 weeks if initially normal.^5^^,^^6^

**Table 1:** Baseline maternal characteristics stratified by type of diabetes.

	Overall	T1DM	T2DM	GDM	Overt DM
N (%)	298	27 (9.1)	87 (29.2)	118 (39.6)	66 (22.1)
Age	34.3 ± 5.5	27.8 ± 6.2	35.3 ± 4.3	35.1 ± 5.3	34.3 ± 5.3
Ethnicity					
Black	281 (94.4)	26 (9.3)	82 (29.2)	108 (38.4)	65 (23.1)
Coloured	3 (1.0)	§0 (0.0)	2 (66.7)	1 (33.3)	0 (0.0)
Indian	7 (2.3)	1 (14.3)	2 (28.6)	4 (57.1)	0 (0.0)
White	7 (2.3)	0 (0.0)	1 (14.3)	5 (71.4)	1 (14.3)
Parity					
≤1	96 (32.2)	17 (17.7)	17 (17.7)	38 (39.6)	24 (25.0)
2	99 (33.2)	8 (8.1)	32 (32.3)	34 (34.3)	25 (25.3)
≥3	103 (34.6)	2 (1.9)	38 (36.9)	46 (44.7)	17 (16.5)
Gravidity					
≤1	21 (7.1)	9 (42.8)	6 (28.6)	5 (23.8)	1 (4.8)
2	40 (13.4)	4 (10.0)	8 (20.0)	11 (27.5)	17 (42.5)
≥3	237 (79.5)	14 (5.9)	73 (30.8)	102 (43.0)	48 (20.3)
Previous miscarriages					
0	182 (61.1)	18 (9.9)	60 (33.0)	61 (33.5)	43 (23.6)
1	80 (26.8)	7 (8.8)	20 (25.0)	36 (45.0)	17 (21.2)
>2	36 (12.1)	2 (5.6)	7 (19.4)	21 (58.3)	6 (16.7)
Gestational age, first visit	23.7 (5.9)	21.7 (4.0)	23.1 (6.3)	24.3 (5.4)	24.2 (6.8)
Gestational hypertension	66 (22.2)	2 (3.0)	20 (30.3)	29 (44.0)	15 (22.7)
Chronic hypertension	35 (11.8)	4 (11.4)	12 (34.3)	11 (31.4)	8 (22.9)
HIV status					
Positive	44 (14.8)	6 (13.6)	17 (38.6)	14 (31.8)	7 (16.0)
ART	40 (13.4)	6 (15.0)	15 (37.5)	12 (30.0)	7 (17.5)

ART, anti-retroviral therapy; DM, diabetes mellitus; GDM, gestational diabetes mellitus; T1DM, type 1 diabetes mellitus; T2DM, type 2 diabetes mellitus. Data expressed as n (%) or mean ± standard deviation (SD).

Patients were seen at the Combined Obstetric-Endocrine Diabetic Clinic every 2 weeks until 32 weeks, then weekly until delivery, typically at 38 weeks. Induction of labour (IOL) was offered unless a caesarean section (CS) was indicated, especially for estimated fetal weights >4,000 g or abdominal circumferences >97%.

Definitions used align with international and local standards: miscarriage is the loss before 22 weeks or <500 g (international), though South African law sets viability at 26 weeks.^12^^,^^13^ Macrosomia is defined as birth weight >4,000 g or >90th percentile, low birth weight (LBW) as <2,500 g, and preterm delivery as birth <37 weeks.^13^ Stillbirth refers to fetal death ≥28 weeks or >500 g, while congenital abnormalities include any intrauterine structural or functional malformations. Advanced maternal age is ≥35 years at delivery.^13^ Severe neonatal hypoglycaemia is <1.65 mmol/L in the first 24 h or <2.5 mmol/L thereafter; RDS is defined by the need for oxygen supplementation to maintain saturation above 85%, subsequently requiring NICU admission.^13^

To evaluate the efficacy of HbA1c and FPG as diagnostic tools for GDM, data from non-GDM patients undergoing OGTT at CMJAH were analysed prospectively. Whole blood samples were collected in sodium fluoride tubes, and glucose levels were measured on the same day using the Cobas 8000 glucose hexokinase method (Roche Diagnostics, Mannheim, Germany). A sub-analysis assessed postpartum dysglycaemia, classifying T2DM, prediabetes, including impaired fasting glucose (IFG) and impaired glucose tolerance (IGT), using both WHO and ADA thresholds (Supplementary Table 3).^5^^,^^6^^,^^14^ Clinical data were extracted from antenatal records, including maternal demographics, pregnancy history, GDM risk factors, treatment and investigations. Anthropometric and blood pressure measurements were conducted by trained diabetes nurses using standard protocols.

Data were cleaned and analysed using R software. Non-parametric tests (Kruskal–Wallis and Wilcoxon rank-sum) compared continuous variables, while χ² or Fisher’s exact tests assessed categorical associations. Logistic regression models evaluated the diagnostic accuracy of HbA1c and FPG, adjusting for covariates. ROC curves were used to determine sensitivity, specificity, and predictive values for HbA1c cut-offs, with optimal thresholds identified via the Youden index. Statistical significance was set at p<0.05.

## Results

A total of 298 participants were included (293 singleton and five twin pregnancies), with GDM being the most common diagnosis (Table 1). Hypertensive disorders were most frequent in women with GDM (39.6%), followed by pre-gestational DM (38.3%) and overt DM (22.1%). The median maternal age was 35 years, with 61.1% classified as advanced maternal age, mainly in the GDM group (46.2%). The average gestational age at first visit was 24 weeks.

Key GDM risk factors included family history, advanced maternal age, obesity and poor obstetric history (Supplementary Tables 4 and 5). Over half of the women with GDM were obese (58.4%), and 63% had a history of macrosomia.

At the first visit, the mean weight was 79.1 kg, with the GDM group showing the highest mean weight (82 kg) and a mean BMI in the obesity range (30.5 kg/m²) (Table 2). Weight gain ranged from 10.5–11% in participants with GDM, overt DM and pre-gestational DM, with the least gain in those with T1DM, though differences were not statistically significant. Despite this, HbA1c levels decreased across all groups except in GDM, where it rose from 5.8% to 6.0% (Table 2). HbA1c at booking was highest in pre-gestational DM, particularly T1DM (8.8%), followed by T2DM (7.8%) and overt DM (7.7%) (Table 2). No significant correlation was found between weight or BMI changes and HbA1c levels (Supplementary Figure 1).

**Table 2:** Change in metabolic parameters from the first visit to delivery.

	Overall	T1DM	T2DM	GDM	Overt DM	p-value
N (%)	298	27 (9.1)	87 (29.2)	118 (39.6)	66 (22.1)	
Weight, baseline (kg)	79.1± 17.1	70 ± 10.4	78.3 ± 17.1	82.0 ± 18.5	78.8 ± 15.3	0.29
Weight, delivery (kg)	86.6 ± 16.6	75.7 ± 10.7	86.4 ± 16.4	89.7 ± 17.4	86.4 ± 15.6	
% change	10.5 ± 0.7	8.5 ± 10.1	11.3 ± 11.3	10.5 ± 12.3	10.4 ± 11.8	
BMI, baseline (kg/m^2^)	29.4 ± 6.6	25.6 ± 3.7	28.7 ± 6.0	30.5 ± 7.3	29.9 ± 6.3	**0.006**
BMI, delivery (kg/m^2^)	32.2 ± 6.6	27.9 ± 5.6	31.6 ± 5.7	33.4 ± 6.8	32.7 ± 6.8	**<0.001**
% change	10.5 ± 11.7	8.5 ± 10.1	11.3 ± 11.3	10.5 ± 12.3	10.4 ± 11.8	0.30
HbA1c, baseline (%)	7.1 ± 2.1	8.8 ± 2.8	7.8 ± 2.5	5.8 ± 0.5	7.7 ± 1.6	**<0.001**
HbA1c, delivery (%)	6.9 ± 1.5	8.4 ± 2.0	7.0 ± 1.3	6.0 ± 0.6	7.0 ± 1.4	**<0.001**
% change	-4.9 ± 16.4	-8.3 ± 16.8	-9.2 ± 17.0	4.2 ± 9.5	-9.7 ± 18.3	**<0.001**

BMI, body mass index; DM, diabetes mellitus; GDM, gestational diabetes mellitus; T1DM, type 1 diabetes mellitus; T2DM, type 2 diabetes mellitus; HbA1c, glycated haemoglobin; SD, standard deviation. Data expressed as n (%) or mean ± standard deviation (SD). Statistical significance p≤0.05, shown in bold.

The mean gestational age at delivery was 37 weeks across all DM groups (Supplementary Table 6). CS was the most common delivery method (67.1%), followed by vaginal delivery (31.5%) and IOL (6.0%), with the GDM group accounting for most IOL cases (Supplementary Table 6). Among 303 births (including twins), polyhydramnios was seen in 6.4%, mainly in the GDM group (Table 3), and preterm delivery occurred in 16%, also predominantly in GDM.

**Table 3:** Distribution of Maternal, fetal and neonatal outcome by glycaemic disorder.

	Overall	T1DM	T2DM	GDM	Overt DM	p-value
N (%)	298	27 (9.1)	87 (29.2)	118 (39.6)	66 (22.1)	
Maternal outcomes						
Polyhydramnios	19 (6.4)	3(15.8)	2(10.5)	8(42.1)	6(31.6)	0.20
Pre-eclampsia	8 (2.7)	1 (12.5)	4 (50.0)	3 (37.5)	0	0.30
Preterm delivery	16 (5.4)	2 (12.5)	3 (18.8)	6 (37.5)	5 (31.2)	0.60
Fetal outcomes, N = 303	Overall		*Early neonatal outcomes, N = 294*			Overall
Stillbirth	9 (2.9)		Neonatal RDS			75 (25.5)
Congenital abnormalities	2 (0.7)		Neonatal hypoglycaemia			134 (45.6)
Macrosomia	27 (8.9)		Low birth weight			55 (18.7)
Miscarriages	7 (2.3)		Birth weight (kg), median (IQR)			3.1 (2.7–3.6)
			APGAR birth, median (IQR)			9 (9–10)
			APGAR 1, median (IQR)			9 (9–10)
			APGAR 2, median (IQR)			10 (10–10)

DM, diabetes mellitus; GDM, gestational diabetes mellitus; T1DM, type 1 diabetes mellitus; T2DM, type 2 diabetes mellitus. IQR, interquartile range; kg, kilogram; RDS, respiratory distress syndrome.

Data expressed as n (%) or median (IQR).

Statistical significance p≤0.05.

Adverse fetal outcomes included macrosomia in 8.9% (n = 27 [T1DM 4, T2DM 13, GDM 6, overt DM 4]), congenital abnormalities (0.7%, n = 2 [T1DM 1 and overt DM 1]), stillbirths (2.9%, n = 9 [T1DM 2, T2DM 3, GDM 4, overt DM]) and miscarriages (2.3%, n = 7 [T1DM 1, T2DM 3, GDM 1, overt 2]), mostly in pre-gestational DM (Table 3). Of 294 live births, four of the five twin pregnancies resulted in only one live birth each and 41.2% required NICU admission. The main complications were neonatal hypoglycaemia in 45.6% (n = 134 (T1DM 10, T2DM 41, GDM 53, overt DM 30)), RDS in 25.5% (n = 75 (T1DM 8, T2DM 21, GDM 27, overt DM 19)), and LBW in 18.7% (n=55 (T1DM 7, T2DM 15, GDM 20, overt DM 13)) (Table 3).

As shown in Supplementary Table 7, one-third of women with pregestational T2DM used metformin pre-pregnancy. During pregnancy, 50% of GDM patients used metformin, and 4% required insulin. When evaluating neonatal outcome stratified by treatment, those with neonatal RDS (n = 75) were predominantly on metformin (lifestyle only (n = 10), metformin (n = 36), insulin (n = 29) and combined insulin and metformin )n = 0)). For those with neonatal hypoglycaemia (n = 134), the distribution of treatment included lifestyle only (n = 22), metformin (n = 60), insulin (n = 50) and combined insulin and metformin (n = 2) and in neonates born with a LBW (n = 55) (lifestyle only (n = 8), metformin (n = 30), insulin (n = 16) and combined insulin and metformin (n = 1)).

**Fig. 1:**
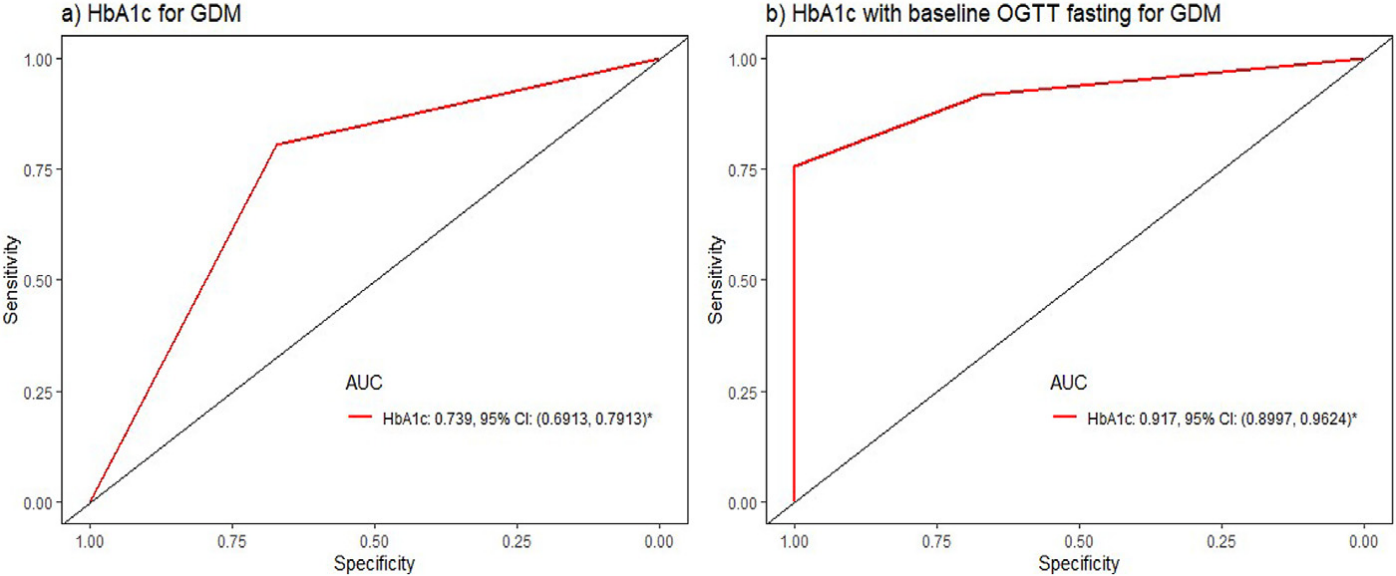
Performance of HbA1c ≥ 5.75% and combined HbA1c ≥ 5.75% & FPG 5.1–6.9 mmol/L for diagnosing GDM. AUC, area under the receiver operating curve; OGTT, oral glucose tolerance test; GDM, gestational diabetes mellitus.

The accuracy of HbA1c for diagnosing GDM was evaluated using logistic regression in 118 GDM and 171 non-GDM patients. An HbA1c ≥ 5.75% combined with FPG 5.1–6.9 mmol/L yielded the highest diagnostic accuracy (AUC: 0.93; sensitivity: 53%; specificity: 91%) (Fig. 1), outperforming HbA1c alone or a 5.5% cutoff (sensitivity: 86%, specificity: 41%) (Supplemental Table 8).

GDM was confirmed in 91.5% of women at the 60-minute OGTT point (Supplemental Table 9). Among 57 women who underwent a 3-month postpartum OGTT, 21% developed DM, 53% had IGT, and IFG was identified in 3.5% (WHO) and 10.5% (ADA) (Supplemental Table 10). Regarding postpartum contraception, 25% chose sterilisation, 62.8% injectables and 11.4% oral contraceptives (Supplemental Table 11).

## Discussion

This study highlights the diagnostic and clinical profile of HFDP in a high-risk South African population. Almost 40% of participants had GDM, and the majority were overweight or obese with additional risk factors such as advanced maternal age. A combined FPG and HbA1c threshold (≥5.75%) performed well in detecting GDM, supporting its utility as a simplified screening tool in low-resource settings where OGTT access is limited. The rates of CS and neonatal complications, including hypoglycaemia and NICU admission, were high, and stillbirths occurred predominantly in those with pre-gestational DM. Notably, over 70% of women with GDM who were followed up had persistent dysglycaemia at 3 months postpartum, with half having IGT and a quarter developing T2DM, highlighting the urgent need for structured postpartum follow-up.

Our study demonstrated a high diagnostic accuracy using combined HbA1c (≥5.75%) and FPG (5.1–6.9 mmol/L), with an AUC of 0.93 and NPV of 95.2%, outperforming pooled data from high-income countries (AUC 0.84)^15^ and regional studies in Nigeria (AUC 0.65)^16^ and South Africa (sensitivity of 29%) for the combined approach.^17^ Given that 90% of GDM cases occur in LMICs, where OGTT use is limited,^17^ alternative screening tools are essential. The STRIDE study showed early pregnancy HbA1c (<16 weeks), with clinical risk factors, could reduce OGTT need by 64%.^18^ Our findings support using combined FPG and HbA1c (≥5.75%) for initial GDM screening in such settings, though its lower sensitivity requires validation in larger cohorts.

This study highlights key risk factors and outcomes in women with GDM and pre-gestational DM. The high prevalence of GDM in our cohort reflects the growing metabolic burden in LMICs and is consistent with local data, possibly attributed to the high incidence of obesity.^19^, ^20^, ^21^ Risk factors such as obesity, advanced maternal age (present in >60%) and family history of DM (70%) were commonly observed, in line with global trends.^20^^,^^21^ Poor obstetric history was reported in 10.2%, reaffirming traditional risk markers.

Hypertensive disorders were common, with chronic hypertension in up to 5% and gestational hypertension in 22%, primarily among those with GDM, potentially reflecting shared insulin resistance and endothelial dysfunction pathways prevalent in LMICs.^19^, ^20^, ^21^ Pre-eclampsia rates were lower than reported elsewhere, possibly due to improved antenatal care.^19^, ^20^, ^21^ Polyhydramnios was predominantly noted in the GDM group, consistent with mid-gestation dysglycaemia.^22^ More than half of the patients had a history of delivering a prior macrosomic infant, with almost one-third of these patients being obese, and over half with GDM, reinforcing obesity’s role in insulin resistance. Macrosomia-related complications such as shoulder dystocia and increased CS delivery further highlight the need for risk factor mitigation.^22^

Vaginal delivery occurred in one-third of overall cases, with induction rates threefold lower in GDM. Mean delivery at 37 weeks deviates from local data as well as national and international guidelines recommending delivery at ≥39 weeks in well-controlled GDM,^5^^,^^14^^,^^19^^,^^22^ possibly reflecting clinician concern over complications amid limited monitoring and glycaemic control. However, delaying delivery to ≥39 weeks is recommended to reduce neonatal respiratory risk. CS rates were high, particularly in GDM, consistent with other South African data,^19^^,^^22^ possibly due to late booking, macrosomia risk and glycaemic concerns. However, macrosomia (>4,000 g) occurred in only 8.9%, with 2% >4,500 g, echoing older local data and suggesting persistent suboptimal glycaemic control or epigenetic influences like fetal hyperinsulinaemia.^23^ Genetic and epigenetic drivers of GDM warrant further study in local populations.^10^

Preterm delivery (5%) was mostly observed in GDM and was lower than typical rates seen in pre-gestational DM,^19^^,^^22^ perhaps reflecting improved antenatal care. In GDM, late diagnosis and poor glycaemic control may still lead to preterm births from risks like macrosomia or preeclampsia.^24^ Stillbirths were more frequent in pre-gestational DM, likely due to chronic hyperglycemia-related vasculopathy impairing placental perfusion via oxidative stress and endothelial dysfunction.^24^ These findings highlight the need for preconception counselling, early glycaemic control and vigilant fetal surveillance. Congenital anomalies were identified in 0.7%, lower than prior local reports, possibly reflecting improved antenatal care and first-trimester glycaemic and folate optimisation.^19^^,^^22^^,^^24^ Miscarriage occurred in 2%, mostly in pre-gestational DM, likely related to vascular damage. However, late booking (median 24 weeks) may underestimate the true rate.^19^^,^^22^

NICU admissions were high, especially in pre-gestational DM, aligning with LMIC and HIC data linking maternal hyperglycaemia to neonatal hypoglycaemia and respiratory distress syndrome (RDS).^25^ Neonatal hypoglycaemia (44.8%) was most common in infants of mothers with T2DM and GDM, potentially driven by fetal hyperinsulinaemia, as supported by existing data.^25^ In pre-gestational DM, elevated RDS is linked to insulin-mediated surfactant suppression, delaying lung maturation.^25^ A recent meta-analysis confirmed that GDM significantly increases the risk of preterm labour (OR 1.51), low 1-minute Apgar scores (OR 1.43), macrosomia (OR 1.70) and CS (OR 1.16), highlighting the importance of close neonatal monitoring in GDM and pre-gestational DM.^25^

There was a high loss to follow-up in our study, largely due to the absence of routine postpartum OGTT scheduling and a dedicated follow-up clinic for GDM patients. In a sub-analysis, three-quarters of women with GDM had persistent dysglycaemia postpartum, with similar performance using ADA and WHO criteria, though ADA’s lower IFG threshold identified more cases.^10^^,^^26^ Over 50% had IGT and 21% developed T2DM, highlighting the need for structured postpartum follow-up to prevent progression to DM.^10^^,^^26^ Differentiating persistent dysglycaemia from undiagnosed pre-gestational T2DM is essential, as up to 50% of women with GDM develop DM within 5 years.^26^^,^^30^ However, postpartum care remains inadequate in LMICs due to fragmented systems and low awareness.^2^ Despite the OGTT being the gold standard at 3–6 months postpartum, uptake is low due to resource constraints and provider oversight.^27^ HbA1c offers greater accessibility, but cannot replace OGTT entirely due to limited sensitivity and specificity.^28^^,^^29^ Alternative approaches include risk stratification using pregnancy OGTT, machine learning to predict follow-up gaps, and integrating postpartum screening with child health visits.^15^^,^^30^ Combining HbA1c with OGTT in early pregnancy may streamline the diagnostic process. For women with HbA1c values between 5.45% and 5.95%, performing an OGTT is recommended for accurate diagnosis,^15^ potentially identifying 86% of GDM cases while reducing OGTT necessity by two-thirds.^15^^,^^30^

This is one of the few studies from LMICs to comprehensively characterise pre-gestational DM and HFDP in a real-world urban African cohort, highlighting both short-term maternal and neonatal outcomes, long-term glycaemic risk and the utility of a simpler, feasible diagnostic tool for GDM.^10^ Study limitations include single-centre design, incomplete neonatal and postpartum data, and the absence of pre-pregnancy weight, BMI or early glycaemic control data.^19^ The small subgroup and low incidence relative to total deliveries introduce potential selection bias. Finally, incomplete postpartum follow-up, with many women lost to care, limits evaluation of long-term glycaemic outcomes.^2^^,^^26^

## Conclusion

This study highlights the urgent need to enhance GDM screening and postpartum follow-up in resource-limited settings. Early diagnosis using combined FPG and HbA1c shows promise as a practical alternative for GDM detection. Strengthening early screening and improving postpartum care may reduce long-term complications for both mothers and their children. Further research is needed to validate these findings in larger populations.

## Ethics approval and consent to participate

Ethical approval was obtained from the University of the Witwatersrand Human Research Ethics Committee (references M170710 and M230675) and the CMJAH Committee. A waiver of informed consent was granted for the retrospective component of the study due to the use of preexisting, de-identified clinical records, which posed minimal risk to participants and made obtaining individual consent unfeasible. Similarly, for the non-GDM cohort, as it involved a sub-analysis of this existing database, informed consent from participants was not required, but a letter of permission from the principal investigator was provided to allow access to de-identified data.

## Funding

This research did not receive any specific grant from funding agencies in the public, commercial, or not-for-profit sectors.

## CRediT authorship contribution statement

**Jaishil Manga:** Writing – original draft, Methodology, Formal analysis, Data curation, Conceptualization. **Natalie Odell:** Writing – review & editing, Supervision, Data curation, Conceptualization. **Lungile Khambule:** Writing – review & editing, Data curation. **Sayuri Harishun:** Writing – original draft, Conceptualization. **Farzahna Mohamed:** Writing – review & editing, Writing – original draft, Supervision, Project administration, Methodology, Formal analysis, Data curation, Conceptualization.

## Declaration of competing interests

The authors declare that they have no known competing financial interests or personal relationships that could have appeared to influence the work reported in this paper.

## Appendix Supplementary materials

Image, application 1
