# Supplementary material for: Hyperglycaemia in pregnancy: Outcomes and diagnostic accuracy of combined modalities
Source: Clin Med (Lond). 2025 Aug 6;25(5):100495. doi: 10.1016/j.clinme.2025.100495 (PMC12395516; doi:10.1016/j.clinme.2025.100495)
Supplement: Supplementary file 1 [file mmc1.docx]

**Supplementary appendix**

**Figures**

Figure 1. Correlation of percentage change in weight and BMI with HbA1c change

Figure 2. Diagnosis of GDM using HbA1c

**Tables**

Table 1. Common risk factors associated with the development of GDM

Table 2. Diagnosis and stratification of hyperglycemia first detected in pregnancy

Table 3. Diagnosis of diabetes, impaired fasting glucose and impaired glucose tolerance

Table 4. Risk factors associated with the development of GDM

Table 5. Maternal risk factors and its association with GDM as compared to no GDM.

Table 6. Distribution of hyperglycemic disorders by gestational age and mode of delivery

Table 7. Diabetic management amongst the various groups

Table 8. Measure of accuracy estimates using multiple logistic regression analysis of HbA1c with FPG for GDM screening.

Table 9 Incidence of GDM as per ADA and WHO criteria at 3 months postpartum.

Table 10. Diagnosis of GDM based on OGTT.

Table 11. Postpartum family planning

**Figures**


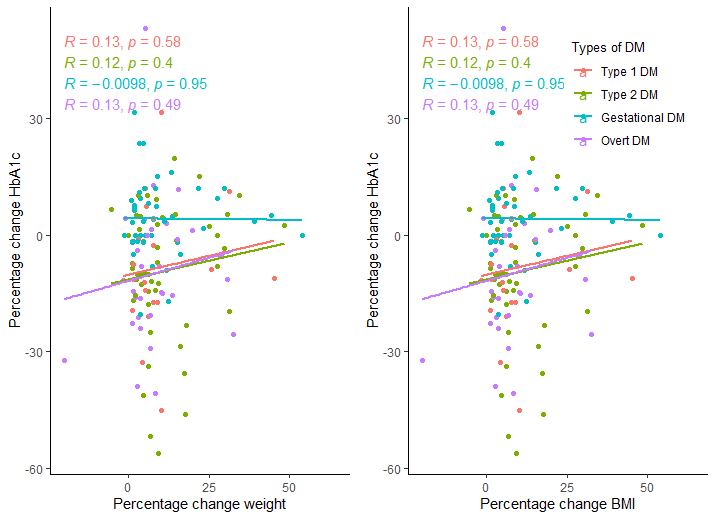


**Figure 1:** Correlation of percentage change in weight and BMI with HbA1c change. Plots of percentage change in weight (left panel) and percentage change of BMI (right panel) by types of DM. Correlation estimates (R), p-value (p).


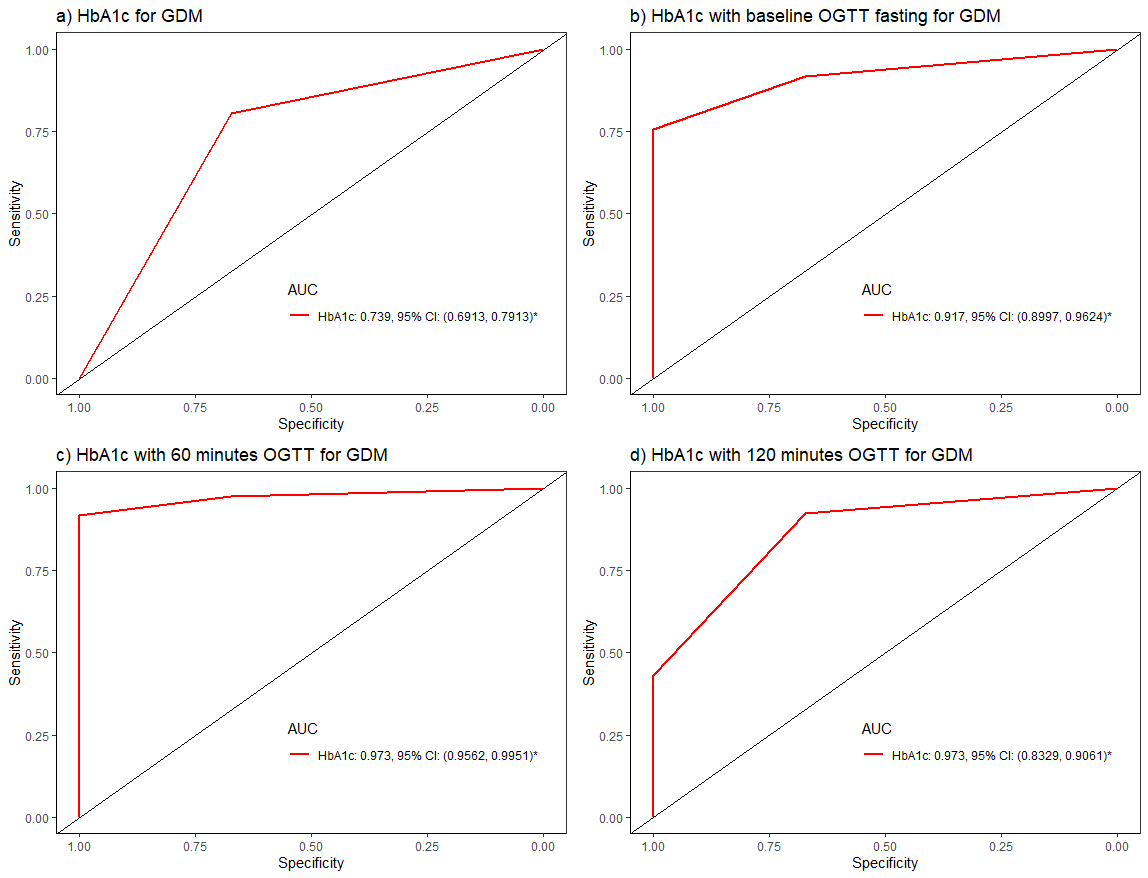


**Figure 2:** Diagnosis of GDM using HbA1c. ROC plots of HbA1c screening for GDM (a) HbA1c default cutpoints (5.50), (b) HbA1c 5.50 with OGTT baseline.

**Table 1. Common risk factors associated with the development of GDM**

| **Risk factors** |
| --- |
| Previous GDM |
| Repeated glycosuria |
| Obesity (BMI > 30 kg/m^2^) |
| Family history of diabetes (first degree relative) |
| Previous history of GDM |
| High risk ethnic groups |
| Previous adverse pregnancy outcomes (macrosomia, stillbirths, recurrent miscarriages, recurrent miscarriages |
| History of polycystic ovarian syndrome |
| **Adapted ADA and SEMDSA guidelines ^[1,5]^** |

**Table 2. Diagnosis and stratification of hyperglycemia first detected in pregnancy**

| **Glucose Test** | **GDM** | **Overt DM** |
| --- | --- | --- |
| Fasting plasma glucose (mmol/L) | ≥ 5.1-6.9 | ≥ 7.0 |
| One hour post glucose load* (mmol/L) | ≥ 10.0 | Not applicable |
| Two hours post glucose load* (mmol/L) | ≥ 8.5 – 11.0 | ≥ 11.0 |
| *****75g Plasma glucose. DM, diabetes mellitus; GDM, gestational diabetes mellitus. **Adapted SEMDSA guidelines^[5]^** | | |

**Table 3. Diagnosis of diabetes, impaired fasting glucose and impaired glucose tolerance**

| **Glucose Test** | **Normal** | **IFG/IGT** | **DM** |
| --- | --- | --- | --- |
| Fasting plasma glucose (mmol/L) | < 5.6 *(DM excluded)* | 6.0-6.9 | ≥ 7.0 |
| Two hour post glucose load* (mmol/L) | < 7.8 *(normal)* | 7.8-10 | ≥ 11.1 |
| HbA1c (%) | < 6.5 *(inconclusive)* |  | ≥ 6.5 |
| Random plasma glucose (mmol/L) | < 5.6 *(DM excluded)* | 5.6-11.0 | ≥ 11.1 |
| *****75g Plasma glucose. IFG, impaired fasting glucose; IGT, impaired glucose tolerance. **Adapted SEMDSA guidelines^[5]^** | | | |

| Table 4. Risk factors associated with development of GDM | | | | | | |
| --- | --- | --- | --- | --- | --- | --- |
| **Risk factor** | **Overall** | **T1DM** | **T2DM** | **GDM** | **Overt DM** | **p-value** |
| Glycosuria | 251 (84.2) | 25 (10.0) | 73 (29.0) | 94 (37.5) | 59 (23.5) | 0.20 |
| Family history | 207 (69.5) | 20 (9.7) | 69 (33.3) | 79 (38.2) | 39 (18.8) | **0.047** |
| Previous GDM | 29 (9.7) | 2 (6.9) | 5 (17.2) | 15 (51.8) | 7 (24.1) | 0.40 |
| Obesity | 89 (29.9) | 0 (0.0) | 21 (23.6) | 52 (58.4) | 16 (18.0) | **0.011** |
| Poor obstetric history | 42 (14.1) | 4 (9.5) | 7 (16.7) | 21 (50.0) | 10 (23.8) | **0.041** |
| Previous macrosomia | 184 (61.7) | 9 (4.9) | 62 (33.7) | 74 (40.2) | 39 (21.2) | **0.005** |
| Advanced maternal age | 182 (61.1) | 8 (4.4) | 60 (33.0) | 75 (41.2) | 39 (21.4) | **0.001** |
| . Data expressed as n (%) or mean ± standard deviation (SD). Statistical significance P≤0.05. | | | | | | |

| **Table 5. Maternal risk factors and its association with GDM as compared to no GDM.** | | | | |  |  |
| --- | --- | --- | --- | --- | --- | --- |
|  | **Overall** | **No GDM *(n=171)*** | **GDM** | **p-value** |  |  |
| ***N (%)*** | 289 | 171 (57.4) | 118 (39.5) |  |  |  |
| **Age, median (IQR)** | 36.0 (32.0, 40.0) | 37.0 (32.0, 40.0) | 35.0 (31.2, 39.0) | 0.20 |  |  |
| **HbA1c, median (IQR)** | 5.5 (5.3, 5.9) | 5.3 (5.0, 5.5) | 5.8 (5.5, 6.0) | **<0.001** |  |  |
| **Parity** |  |  |  |  |  |  |
| 1  2  3 | 64 (34.4) | 26 (40.6) | 38 (59.4) | 0.50 |  |  |
|  | 55 (29.6) | 21 (38.2) | 34 (61.8) |  |  |  |
|  | 67 (36.0) | 21 (31.3) | 46 (68.7) |  |  |  |
| **Gravida** |  |  |  |  |  |  |
| 1  2  3 | 8 (4.3) | 3 (37.5) | 5 (62.5) | 0.50 |  |  |
|  | 21 (11.3) | 10 (47.6) | 11 (52.4) |  |  |  |
|  | 157 (84.4) | 55 (35.0) | 102 (65.0) |  |  |  |
| **Previous lost pregnancies**  0  1  >2 |  |  |  |  |  |  |
|  | 99 (53.2) | 38 (38.4) | 61 (61.6) | 0.90 |  |  |
|  | 55 (29.6) | 19 (34.5) | 36 (65.5) |  |  |  |
|  | 32 (17.2) | 11 (34.4) | 21 (65.6) |  |  |  |
| **Repeated glycosuria** | 94 (50.3) | 0 (0.0) | 94 (100.0) | **<0.001** |  |  |
| **Previous GDM** | 15 (8.0) | 0 (0.0) | 15 (100.0) | **0.002** |  |  |
| **Previous miscarriage** | 29 (15.5) | 28 (96.6) | 1 (3.4) | **<0.001** |  |  |
| **Advanced maternal age** | 117 (67.4) | 42 (35.9) | 75 (64.1) | 0.150 |  |  |
| **BMI Categories** |  |  |  |  |  |  |
| Normal  Overweight  Obese | 45 (24.3) | 9 (20.0) | 36 (80.0) | **0.009** |  |  |
|  | 44 (23.8) | 14 (31.8) | 30 (68.2) |  |  |  |
|  | 96 (51.9) | 44 (45.8) | 52 (54.2) |  |  |  |
| **HIV status** | 31 (16.8) | 17 (54.8) | 14 (45.2) | **0.018** |  |  |
| GDM, gestational diabetes mellitus; HIV, human immune-deficiency virus. . Data expressed as n (%), mean ± standard deviation (SD) or median (IQR). Statistical significance P≤0.05. | | | | |  |  |

| Table 6. Distribution of hyperglycemic disorders by gestational age and mode of delivery | | | | | | |
| --- | --- | --- | --- | --- | --- | --- |
| **Mode of Delivery** | **Overall** | **T1DM** | **T2DM** | **GDM** | **Overt DM** | **p-value** |
| ***N (%)*** | 298 | 27 (9.1) | 87 (29.2) | 118 (39.6) | 66 (22.1) |  |
| Gestational age | 37.3 (1.9) | 36.9 (2.7) | 37.5 (1.7) | 37.3 (1.8) | 37.2 (1.8) | 0.54 |
| Cesarean section | 200 (67.1) | 18 (9.0) | 60 (30.0) | 74 (37.0) | 48 (24.0) | 0.50 |
| Vaginal | 94 (31.5) | 9 (9.6) | 27 (28.7) | 42 (44.7) | 16 (17.0) | 0.50 |
| DM, diabetes mellitus; GDM, gestational diabetes mellitus; T1DM, type 1 diabetes mellitus; T2DM, type 2 diabetes mellitus. Data expressed as n (%)or mean ± standard deviation (SD).. Statistical significance P≤0.05. | | | | | | |

**Table 7. Diabetic management amongst the various groups**

| **Characteristics** | **Overall** | **Type 1** | **Type 2** | **GDM** | **Overt** | **p-value** |
| --- | --- | --- | --- | --- | --- | --- |
| ***N (%)*** | 298 | 27 (9.1) | 87 (29.2) | 118 (39.6) | 66 (22.1) |  |
| **Previous treatment types** |  |  |  |  |  |  |
| No Treatment | 3 (1.0) | 0 (0.0) | 0 (0.0) | 2 (66.7) | 1 (33.3) | **<0.001** |
| Diet only | 41 (13.8) | 0 (0.0) | 3 (7.3) | 37 (90.2) | 1 (2.5) |  |
| Metformin | 154 (51.7) | 1 (0.6) | 48 (31.2) | 75 (48.7) | 30 (19.5) |  |
| Insulin | 100 (33.6) | 26 (26.0) | 36 (36.0) | 4 (4.0) | 34 (34.0) |  |
| **Treatment types during pregnancy** | | | | | | |
| No Treatment | 2 (0.7) | 1 (50.0) | 0 (0.0) | 0 (0.0) | 1 (50.0) | **<0.001** |
| Diet only | 40 (13.4) | 0 (0.0) | 2 (5.0) | 38 (95.0) | 0 (0.0) |  |
| Metformin | 152 (51.0) | 0 (0.0) | 45 (29.6) | 76 (50.0) | 31 (20.4) |  |
| Insulin | 98 (32.9) | 26 (26.5) | 36 (36.7) | 4 (4.1) | 32 (32.7) |  |
| Insulin and Metformin | 6 (2.0) | 0 (0.0) | 4 (66.7) | 0 (0.0) | 2 (33.3) |  |
| DM, diabetes mellitus; GDM, gestational diabetes mellitus; T1DM, type 1 diabetes mellitus; T2DM, type 2 diabetes mellitus. Data expressed as n (%). Statistical significance P≤0.05. | | | | | | |

**Table 8. Measure of accuracy estimates using multiple logistic regression analysis of HbA1c with FPG for GDM screening**

|  |  |  | **Measures of accuracies** | | | |
| --- | --- | --- | --- | --- | --- | --- |
| **Variable**  **(Cutoff)** | **Added OGTT variable** | **AUC** | **Sensitivity** | **Specificity** | **PPV** | **NPV** |
| **HbA1c (5.50)** | HbA1c with FPG baseline | 0.918 | 0.8571 | 0.4086 | 0.7781 | 0.5416 |
|  | HbA1c with 60 min | 0.974 | 0.8198 | 0.5153 | 0.8037 | 0.5416 |
|  | HbA1c with 120 min | 0.869 | 0.7308 | 0.8346 | 0.9145 | 0.5616 |
| **HbA1c (5.75)** | HbA1c with FPG baseline | 0.931 | 0.5275 | 0.9131 | 0.6046 | 0.9528 |
|  | HbA1c with 60 min | 0.976 | 0.5315 | 0.9005 | 0.5882 | 0.9497 |
|  | HbA1c with 120 min | 0.834 | 0.5577 | 0.8928 | 0.6019 | 0.9972 |
| FPG, fasting plasma glucose; HbA1c, glycated hemoglobin; OGTT, oral glucose tolerance test; NPV, negative predictive value; PPV, positive predictive value. | | | | | | |

**Table 9. Diagnosis of GDM based on OGTT.**

| **OGTT** | **Overall** | **No GDM** | **GDM** |
| --- | --- | --- | --- |
| ***N (%)*** | 289 | 171 (57.4) | 118 (39.5) |
| **GDM fasting at baseline** |  |  |  |
|  | 200 (69.2) | 171 (100) | 29 (24.6) |
| Present | 89 (30.8) | 0 (0.0) | 89 (75.4) |
| **GDM at 60 minutes** |  |  |  |
|  | 181 (62.6) | 171 (100) | 10 (8.5) |
| Present | 108 (37.4) | 0 (0.0) | 108 (91.5) |
| **GDM at 120 minutes** |  |  |  |
|  | 238 (82.4) | 171 (100) | 67 (56.8) |
| Present | 51 (17.6) | 0 (0.0) | 51 (43.2) |

GDM, gestational diabetes mellitus; OGTT, oral glucose tolerance test. Data expressed as n (%).

**Table 10. Incidence of persistent glucose abnormalities at 3 months postpartum in women diagnosed with GDM as per ADA and WHO criteria.**

|  | | **Overall, *n = 57*** |
| --- | --- | --- |
| **Criteria** | | **n (%)** |
| **Postpartum OGTT SEMDSA^[5]^** | NIL | 13 (22.8) |
|  | IFG | 2 (3.5) |
|  | IGT | 30 (52.6) |
|  | DM | 12 (21.1) |
| **Postpartum OGTT ADA^[14]^** | NIL | 9 (15.8) |
|  | IFG | 6 (10.5) |
|  | IGT | 30 (52.6) |
|  | DM | 12 (21.1) |
| ADA, American Diabetes Association; DM, diabetes mellitus; IFG, impaired fasting glucose; IGT, impaired glucose tolerance test; OGTT, oral glucose tolerance test; Data expressed as n (%). | | |

**Table 11. Postpartum family planning.**

| **Characteristics** | **Overall** | **Type 1** | **Type 2** | **GDM** | **Overt DM** | **p-value** |
| --- | --- | --- | --- | --- | --- | --- |
| ***N (%)*** | 298 | 27 (9.1) | 87 (29.2) | 118 (39.6) | 66 (22.1) |  |
| **Barrier contraception** | 206 (69.1) | 24 (11.7) | 62 (30.1) | 79 (38.3) | 41 (19.9) | 0.069 |
| **Sterilization** | 78 (26.2) | 3 (3.9) | 20 (25.6) | 34 (43.6) | 21 (26.9) | 0.20 |
| **Oral** | 34 (11.4) | 2 (5.9) | 8 (23.5) | 16 (47.1) | 8 (23.5) | 0.80 |
| **Injectable** | 187 (62.8) | 22 (11.8) | 61 (32.6) | 67 (35.8) | 37 (19.8) | **0.028** |

DM, diabetes mellitus; GDM, gestational diabetes mellitus; T1DM, type 1 diabetes mellitus; T2DM, type 2 diabetes mellitus. Data expressed as n (%) or mean ± standard deviation (SD). Statistical significance P≤0.05.
